# 

**Published:** 1888-10

**Authors:** 


### TABLE OF CONTENTS.

ORIGINAL AND SELECTED ARTICLES.

Aseptic, antiseptic and septic surgery, by J. McF. Gaston, M. D., Professor of Principles and Practice of Surgery ........ 361
"The pearl upon the rose," by Henry A. Du Bois, Ph B., M.D, San Rafael, Cal ........ 372
Distilled tar water in the treatment of hemorrhages ........ 376

ABSTRACTS AND GLEANINGS.

Summer diarrhœas of infancy and childhood, by Louis Starr, M. D., Philadelphia ........ 377
Antiseptic action of chloroform water ........ 380
Etiology and treatment of yellow fever ........ 381
The boracic-acid leucorrhœa treatment ........ 382
Treatment of carcinoma uteri ........ 383
Saccharin ........ 384
Treatment of dysentery; carbolic acid in treatment of typhoid fever ........ 385
Antipyrin in the treatment of seminal emissions; one-child sterility ........ 386
Photoxylin; opium habit and menstruation ........ 387
Antiseptic treatment of typhoid; the prevention of ophthalmia neonatorum; the treatment of hybrocele ........ 388
Glycerine suppositories for habitual constipation; yellow fever ........ 389
Rules for bathers, oxycyanide of mercury the best of antiseptics; acetanilide—antifebrin ........ 390

SCIENTIFIC ITEMS.

American Association for the advancement of Science; ants and their ways ........ 391
Ostriches; snake bite and yellow fever; fire and water-proof paper ........ 392

PRACTICAL NOTES AND FORMULÆ.

Nervous headaches; formula for antifebrine; formula for dysmenorrhœa; acute tonsilitis; for diphtheria ........ 393
Removing indelible ink; napthaline in dysentery; hypodermic injections in malarial attacks; prescription for headache; digitalis as a diuretic and laxative ........ 394

EDITORIALS AND MISCELLANEOUS.

Editorial brevities ........ 395
Prof. Gaston's introductory ........ 396
Professional dishonor; a noble institution ........ 397
Letter from New York—yellow fever prevented by innoculation ........ 398
Special notes ........ 400

